# Veterinary Medical Society of the University of Pennsylvania

**Published:** 1897-03

**Authors:** John E. Spindler

**Affiliations:** Secretary


### VETERINARY MEDICAL SOCIETY OF THE UNIVERSITY OF PENNSYLVANIA.

THE regular bimonthly meeting was held on Friday evening, February 5, 1897, in the lower lecture-room. Meeting called to order at 8 P.M.

After roll-call and reading of the minutes of previous meeting there was an inaugural address by President F. Klein, and the Secretary read an account of a collection of monstrosities.

There were two referred questions, as follows: One by Mr. William Shaw, class '97: "Resolved, That our patrons and visitors should not be admitted to the hospital or clinics;" the other by Mr. J. Repp, class '98: "On the Manufacture and Therapeutic Action of Sodium Chloride."

The debate of the evening: "Resolved, That Fast Horses have been Detrimental to Mankind." Disputants, affirmative—Messrs. White, class '97; Blount, class '98; Keiter, class '99; negative—Messrs. Lushington, class '97; Jones, class '98; Fox, class '99. The judges were Messrs. Ray Barber, class '97; Al. Cunningham, class '98; J. P. Miller, class '99, whose decision was two in favor of negative and one in favor of affirmative side of the question.

The Society decided to subscribe for the *Pennsylvanian* after hearing a favorable report by critic Wm. Shaw.

Adjourned at 10.30 P.M.

The bimonthly meeting was called to order by President Klein at 7. 45, Friday evening, February 19, 1897. All members responded to roll-call.

Motion to suspend the regular order of business for Dr. Ravenal's lecture was carried. Dr. Ravenal discussed the history of bacteriology and its promoters, among whom was the great Pasteur; he ably discussed all points of advancement made by Pasteur and the bacteriology of to-day, and the future prospects.

Next in order was a debate: "Resolved, that the veterinarian should not compound his own prescriptions." Disputants, affirmative: Cunningham, Townsend, and Barber; negative: Hernschein, Jones, and Hoopes. Judges: Ranck, Spaeth, and Keiter, whose decision was two affirmatives to one negative. Decision of house, negative.

Following this Mr. Montague answered ably to the referred question, "What is the Chief Characteristic of the Percheron Horse?" Critic Lushington's report was spicy and encouraging.

After business of minor importance the Society adjourned to meet March 5, 1897.

JOHN E. SPINDLER,
Secretary,

